# Catechin and Phenolic Profiles of Fermented Miang (*Camellia sinensis* var. *assamica*) and Their Application as Natural Antioxidants in Cosmetic Formulations

**DOI:** 10.3390/antiox15040497

**Published:** 2026-04-16

**Authors:** Panee Sirisa-Ard, Jakaphun Julsrigival, Sunee Chansakaow, Suchart Punjaisee, Pramote Tipduangta, Kiatisak Pholsongkram, Wannaree Charoensup, Nichakan Peerakam, Nararat Akarchariya

**Affiliations:** 1 Department of Pharmaceutical Sciences, Faculty of Pharmacy, Chiang Mai University, Chiang Mai 50200, Thailand; 2 Cluster of Excellence on Biodiversity Based Economics and Society (B.BES-CMU), Chiang Mai University, Chiang Mai 50200, Thailand; 3 Innovation Center for Holistic Health, Nutraceuticals and Cosmeceuticals, Faculty of Pharmacy, Chiang Mai University, Chiang Mai 50200, Thailand; 4 Department of Medical Technology, Faculty of Associated Medical Sciences, Chiang Mai University, Chiang Mai 50200, Thailand; 5 Department of Food Science and Technology, Faculty of Science, Payap University, Chiang Mai 50000, Thailand; 6 Division of Pharmacognosy and Pharmaceutical Chemistry, Faculty of Pharmaceutical Sciences, Burapha University, Chon Buri 20131, Thailand; 7 Medicinal Plant Innovation Center, Faculty of Pharmacy, Chiang Mai University, Chiang Mai 50200, Thailand

**Keywords:** fermented Miang, *Camellia sinensis* var. *assamica*, catechins, phenolics, antioxidant, probiotic cosmetics, formulation stability

## Abstract

Fermented Miang (*Camellia sinensis* var. *assamica*) serves as a valuable source of bioactive polyphenols and probiotic-associated components. This study characterized the catechin composition of fermented Miang extracts and evaluated their antioxidant capacity and suitability for cosmetic formulations. High-performance liquid chromatography (HPLC) analysis showed that epigallocatechin gallate (EGCG) was the predominant catechin (7.00 ± 0.93 mg/g dry weight), followed by catechin (C), epicatechin (EC), epicatechin gallate (ECG), and epigallocatechin (EGC). The extracts remained physically and chemically stable for at least three months under various storage conditions, with the dried extract form offering advantages for handling and formulation. Fermentation duration significantly influenced phenolic accumulation and antioxidant activity, with four-month fermentation showing the highest activity. Prototype cleansing formulations, including transparent/opaque soap bars, liquid soap, and shampoo containing fermented Miang extract, exhibited acceptable physicochemical characteristics and retained antioxidant function. These findings highlight fermented Miang as a promising natural ingredient for antioxidant and probiotic-inspired cosmetic applications.

## 1. Introduction

Natural products have long been incorporated into cosmetic formulations due to their broad biological activities, relatively low toxicity, and high consumer acceptance as sustainable plant-derived ingredients. In particular, plant-derived antioxidants have attracted increasing attention for their ability to neutralize reactive oxygen species (ROS), which play a key role in skin aging, inflammation, and environmental stress–induced damage. Phenolic compounds such as flavonoids, catechins, and phenolic acids are among the most effective natural antioxidants and are widely used in dermatological and cosmeceutical applications due to their radical-scavenging activity and protective effects against oxidative stress and photoaging [1,2,3,4].

Tea plants (*Camellia sinensis*) represent one of the richest natural sources of polyphenolic antioxidants, especially catechins. Major catechins found in tea include epigallocatechin gallate (EGCG), epigallocatechin (EGC), epicatechin (EC), and epicatechin gallate (ECG), which collectively contribute to the antioxidant, anti-inflammatory, and photoprotective activities of tea-derived extracts [4,5]. These compounds have been shown to modulate multiple skin-related biological pathways, including the inhibition of matrix metalloproteinases, stimulation of collagen synthesis, and reduction of ultraviolet-induced oxidative damage. Consequently, tea extracts have been increasingly explored as active ingredients in cosmetic formulations aimed at preventing skin aging and improving skin barrier function.

In Northern Thailand, *Camellia sinensis* var. *assamica* is traditionally processed into a fermented tea product known as Miang. This traditional product is produced through anaerobic fermentation of tea leaves and has been consumed locally for centuries. During fermentation, microbial activity modifies the biochemical composition of the tea leaves, leading to changes in phenolic profiles, enhancement of antioxidant properties, and the generation of microbial metabolites. Fermented Miang has also been reported to contain various species of lactic acid bacteria, particularly *Lactobacillus* spp., which may contribute to probiotic-associated health benefits and improved bioavailability of bioactive compounds [6,7].

Recent developments in cosmetic science have highlighted the potential role of microbial-derived components including probiotics, postbiotics, and microbial lysates in improving skin health. Such ingredients may support skin barrier function, modulate the skin microbiome, and reduce inflammation associated with aging and environmental stress. Consequently, fermented plant materials rich in both phytochemicals and beneficial microorganisms have emerged as promising candidates for the development of next-generation cosmeceuticals and probiotic-inspired skincare products [8].

Despite the long-standing traditional use of fermented Miang, systematic investigations into its phytochemical composition, antioxidant capacity, and potential applications in cosmetic formulations remain limited. In particular, the relationship between fermentation duration, catechin composition, antioxidant activity, and formulation stability has not been fully investigated. Understanding these factors is essential for evaluating fermented Miang as a potential source of bioactive ingredients for cosmetic and dermatological applications.

Therefore, the objectives of the present study were to (i) characterize the catechin profile of fermented Miang extracts using high-performance liquid chromatography (HPLC), (ii) evaluate the total phenolic content (TPC), total flavonoid content (TFC), and antioxidant activity of the extracts in relation to fermentation time, (iii) determine the presence of *Lactobacillus* species in fermented Miang samples, and (iv) develop and assess prototype cosmetic formulations containing fermented Miang extract. The findings of this study aim to provide scientific evidence supporting the utilization of fermented Miang as a natural antioxidant and probiotic-inspired ingredient for cosmetic applications. This study is the first to systematically integrate fermentation time optimization, catechin profiling using HPLC, probiotic-associated *Lactobacillus* characterization, and application in cosmetic formulations with stability evaluation. Unlike previous studies focusing only on phytochemical or microbial aspects, this work establishes a direct relationship between fermentation duration, bioactive compound profiles, antioxidant activity, and formulation performance, thereby providing a comprehensive framework for developing fermented plant-based cosmeceuticals.

## 2. Materials and Methods

### 2.1. Materials

Fermented Miang samples were sourced from Pa-Miang and Mae Jam villages (Lampang Province, Thailand). The use of mature tea leaves was selected based on their higher polyphenol content and traditional use in Miang production, which has been reported to influence catechin yield and fermentation behavior. Reagents included aluminum chloride, 2,2-diphenyl-1-picrylhydrazyl (DPPH), ethanol, Folin–Ciocalteu reagent, gallic acid, methanol, propylene glycol, quercetin, sodium carbonate, sodium hydroxide, sodium nitrite were purchased from Merck (Bangkok, Thailand). Acetonitrile, phosphoric acid and tetrahydrofuran were purchased from RCI Labscan (Bangkok, Thailand). Soap base, oils and surfactants were purchased from Union Science (Chiang Mai, Thailand).

### 2.2. Extraction of Fermented Miang for Cosmetics

FM samples were fermented for 2, 4, or 6 months at 25 ± 2 °C, 60–70% RH. Dried FM powder (100 g) was macerated with propylene glycol:water (6:4, *v*/*v*; 500 mL). Extracts showing the highest antioxidant activity were advanced to formulation and physicochemical testing.

### 2.3. HPLC Determination of Catechins

The quantification of catechins in fermented Miang extracts was performed using a High-Performance Liquid Chromatography (HPLC) system, following the modified method of Masa and Vilanova [9]. Dried fermented Miang (FM) samples were ground using a hammer mill (Crompton, Model 2000 Series, Essex, UK) to pass through a 1.2 mm sieve.

Catechin and catechin-derivative contents were determined using an HPLC system (Shimadzu Scientific Instruments, Kyoto, Japan) consisting of an SCL-10A system controller, GT-154 degasser, FCV-10AL mixer, LC-10AD liquid chromatography pump, SPD-10A UV–VIS detector, CTO-10Avp column oven, and CBM-10A communications bus module. A C18 reversed-phase column (4.6 mm × 250 mm; Waters, Wexford, Ireland) was employed for separation.

The mobile phases consisted of:

Mobile phase A: 0.2% (*v*/*v*) phosphoric acid (86.5% *v*/*v*), acetonitrile (12% *v*/*v*), and tetrahydrofuran (1.5% *v*/*v*).

Mobile phase B: 0.2% (*v*/*v*) phosphoric acid (73.5% *v*/*v*), acetonitrile (25% *v*/*v*), and tetrahydrofuran (1.5% *v*/*v*).

A linear gradient elution program was applied as follows: 0–30 min, 0–100% of mobile phase A; followed by a 10 min gradual increase in mobile phase B to 100%, and holding for 20 min. The flow rate was maintained at 1.0 mL/min. After elution, the system was returned to the initial condition (100% A).

Detection was performed at 280 nm and 210 nm. The column oven temperature was 25 °C. Quantification was achieved by comparing the peak areas of the samples with those of standard catechin compounds: epigallocatechin (EGC), catechin (C), epicatechin (EC), epigallocatechin gallate (EGCG), and epicatechin gallate (ECG). Calibration curves were constructed using these reference standards to determine the concentrations (mg/g dry weight) of each catechin derivative.

### 2.4. Total Phenolic Content (TPC)

The total phenolic content (TPC) of the fermented Miang extracts was determined using the Folin–Ciocalteu colorimetric method, with gallic acid as the reference standard, according to the procedure described by Velioglu et al. [10] with slight modifications.

Briefly, 1.0 mL of the extract was diluted to 10.0 mL with distilled water, and a 0.5 mL aliquot of this diluted extract was further diluted to 10.0 mL. Then, 1.0 mL of the final diluted sample was transferred into a test tube (in triplicate) and mixed with 5.0 mL of a tenfold-diluted Folin–Ciocalteu reagent (1:10 *v*/*v* in water). Then, 4.0 mL of sodium carbonate solution (7.5% *w*/*v*) was added. The reaction mixture was incubated at room temperature for 2 h in the dark.

The absorbance of the samples was measured at 765 nm using a UV–VIS spectrophotometer, with distilled water as the blank. Gallic acid standard solutions (2–40 µg/mL) were prepared in methanol to generate a calibration curve.

TPC values were calculated from the calibration equation and expressed as milligrams of gallic acid equivalents per 100 mL of extract (mg GAE/100 mL) using the following formula:

TPC (mg GAE/100 mL) = (C × V)/m

where C is the concentration from calibration curve, V is volume, and m is sample weight.

All measurements were performed in triplicate, and the results were expressed as the mean ± standard deviation (SD).

### 2.5. Total Flavonoid Content (TFC)

The total flavonoid content (TFC) of the fermented Miang extracts was determined using the aluminum chloride colorimetric method as described by Hashish et al. [11], with slight modifications.

Briefly, 1.0 mL of each extract was diluted to 20.0 mL with distilled water. An aliquot of 1.0 mL of the diluted extract was transferred into a 10 mL volumetric flask, followed by the addition of 4.0 mL of distilled water and 0.5 mL of sodium nitrite solution (5% *w*/*v*). After standing for 5 min, 0.5 mL of aluminum chloride solution (10% *w*/*v* in ethanol) was added. At the 6th minute, 2.0 mL of sodium hydroxide solution (1 M) was added, and the final volume was adjusted to 10 mL with distilled water.

The solution was mixed thoroughly, and the absorbance was measured at 510 nm using a UV–VIS spectrophotometer. Quercetin was used as the reference standard. Calibration curves were constructed using quercetin standard solutions (5–40 µg/mL). The total flavonoid content of the extracts was expressed as milligrams of quercetin equivalents per 100 mL of extract (mg QE/100 mL).

All determinations were carried out in triplicate, and results were expressed as the mean ± standard deviation (SD).

### 2.6. DPPH Radical Scavenging

The antioxidant activity of the fermented Miang extracts was evaluated using the 2,2-diphenyl-1-picrylhydrazyl (DPPH) radical scavenging assay, as described by Wojdylo et al. [12] with slight modifications. This assay is widely used to assess the free radical scavenging potential of bioactive compounds in plant extracts.

A DPPH stock solution was prepared in methanol (81.2 µM). One milliliter of the sample extract was mixed with 5.0 mL of the DPPH solution and vigorously shaken. The reaction mixture was incubated in the dark at room temperature (25 ± 2 °C) for 30 min. The absorbance of the mixture was measured at 517 nm using a UV–VIS spectrophotometer, with methanol as the blank.

DPPH radical scavenging activity (%) was calculated according to the following equation:

DPPH radical scavenging activity (%) = [(*A*_control—*A*_sample)/*A*_control] × 100

where *A*_control is the absorbance of the DPPH solution without extract, and *A*_sample is the absorbance of the DPPH solution containing the sample extract.

The extract concentration providing 50% inhibition (IC_50_) was determined graphically by plotting the percentage inhibition against sample concentration. All measurements were performed in triplicate, and the results were expressed as the mean ± standard deviation (SD).

### 2.7. Lactobacillus Enumeration and Identification

The presence and enumeration of *Lactobacillus* species in fermented Miang (FM) samples were determined using the methods described in the Bacteriological Analytical Manual (BAM) [13] and the AOAC Official Methods [14], with slight modifications.

For bacterial enumeration, 10 g of each homogenized sample was aseptically transferred into a 250 mL Erlenmeyer flask containing 90 mL of Tryptic Soy Broth (TSB), yielding a 1:10 dilution. 10-fold serial dilutions were prepared up to 10^−7^ by transferring 1 mL of each dilution into 9 mL of TSB and mixing thoroughly with a vortex mixer.

An aliquot of 0.1 mL from each dilution was spread on the surface of de Man, Rogosa, and Sharpe (MRS) agar plates containing bromocresol purple as a pH indicator. All plates were incubated at 35 ± 2 °C for 48 h under aerobic conditions. After incubation, colonies showing characteristic *Lactobacillus* morphology were counted on plates containing 25–250 colonies. The average colony count was calculated, and results were expressed as colony-forming units per gram of sample (CFU/g).

Representative colonies with distinct morphologies were subcultured into Thioglycolate Broth and incubated at 35 ± 1 °C for 24 h. Pure cultures were obtained by re-streaking on fresh MRS agar plates. Identification of *Lactobacillus* species was performed using biochemical characterization with the API 50 CHL^®^ test kit (bioMérieux, Craponne, France). The test results were interpreted according to the manufacturer’s instructions to confirm the presence of *Lactobacillus* strains in the FM samples.

### 2.8. Cosmetic Prototypes and Stability Testing

FM extract (5% *w*/*w*) was incorporated into transparent/opaque soap bars, liquid soap, and shampoo (Table 1, Table 2, Table 3 and Table 4). The stability study of the developed formulations was conducted following a scale-up of production and subsequent packaging into suitable containers. The products were subjected to accelerated stability testing at 4 °C, RT, and 45 °C for up to 3 months. Additionally, cyclic temperature stress tests were performed by alternating storage at 4 °C and 45 °C every 24 h for 1 and 5 cycles (H&C condition).

During the stability testing period, all formulations were evaluated for physical and physicochemical parameters, including phase separation, color change, pH, and viscosity. In addition, antioxidant activity of each product was assessed to determine the retention of bioactive properties after storage.

### 2.9. Statistics

Statistical analysis was performed using GraphPad Prism software (GraphPad Prism version 8.0.2, GraphPad Software Inc., San Diego, CA, USA). All experiments were conducted in triplicate, and the results are expressed as the mean ± standard deviation (SD). One-way analysis of variance (ANOVA), followed by Tukey’s multiple comparison post hoc test (*p* < 0.05), was used to determine statistically significant differences among groups. For pairwise comparisons, the Bonferroni test (*p* < 0.05) was applied where appropriate.

## 3. Results and Discussion

### 3.1. Fermentation Time Effects and Extract Stability

Fermented Miang (FM) samples were prepared using mature tea leaves fermented for 2, 4, and 6 months. Among these, the 4-month fermented sample (FMB) exhibited the highest levels of bioactive compounds and antioxidant activity. Specifically, FMB showed a total phenolic content (TPC) of 263.22 ± 2.01 mg GAE/100 g dry weight, total flavonoid content (TFC) of 74.66 ± 2.65 mg QE/100 g dry weight, an IC_50_ value of 4.44 ± 0.00 mg/mL, and 15.62 ± 0.04% inhibition of DPPH radicals (Table 5). These findings indicate that a four-month fermentation period provides optimal conditions for the accumulation of phenolic and flavonoid compounds, resulting in enhanced antioxidant potential compared with 2- and 6-month fermentations.

The extract yield of FMB was 57.50 ± 10.78%, with a pH of 6.02 ± 0.03. The extract appeared as a clear yellow-to-orange-brown solution with a characteristic fermented odor and no visible precipitation. Both TPC and TFC were positively correlated with the antioxidant activity of the extract, confirming that polyphenolic constituents were the main contributors to the observed activity.

The three-month stability study of FMB extracts under different storage conditions is summarized in Table 6. FMB remained stable across all temperature conditions, particularly at 4 °C, maintaining 13.62 ± 0.26% inhibition of DPPH activity. The differences in inhibition among the four storage conditions were negligible, which may be attributed to the strong antioxidant capacity of the FMB extract. Furthermore, the pH values showed minimal variation throughout the testing period, suggesting that catechin derivatives in FMB were relatively stable when stored at low temperature, protected from light, and under minimal oxidation [15]. The enhanced antioxidant activity observed in FMB may be attributed to microbial-mediated biotransformation of polyphenolic compounds during fermentation. Enzymatic activities can release bound phenolics from plant matrices, thereby increasing the concentration of free and more bioavailable phenolic compounds [16]. In addition, oxidoreductase-mediated reactions and microbial metabolism can modify catechin structures, leading to the formation of derivatives with altered redox potential and enhanced antioxidant properties. Fermentation has also been reported to increase total phenolic content and antioxidant capacity through the liberation of bound phenolics and enhancement of bio accessibility [17]. Furthermore, lactic acid bacteria play a crucial role in transforming polyphenols into smaller metabolites with improved solubility, stability, and biological activity, which contributes to enhanced antioxidant performance [18]. These biochemical transformations likely explain the superior antioxidant activity observed in the 4-month fermented sample, representing an optimal balance between compound release and degradation.

The FMB extract, due to its superior stability and high antioxidant activity, was selected for incorporation into cosmetic formulations as a biologically active ingredient. Similar findings have been reported for flavonoids, alkaloids, and phenolic acids in green tea formulations, which demonstrated improved stability under mildly acidic conditions [19].

### 3.2. Catechin Profiles by HPLC

**Figure 1:**
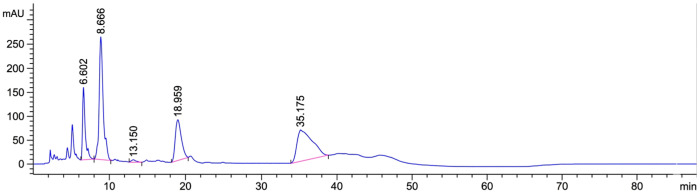
HPLC chromatogram of fermented Miang extract (FMB).

Analysis of bioactive compounds in fermented Miang (FMB) leaves using HPLC revealed that epigallocatechin gallate (EGCG) was the most abundant catechin, with a concentration of 7.00 ± 0.93 mg/g dry weight, followed by catechin (C) (2.41 ± 0.02 mg/g), epicatechin (EC) (2.23 ± 0.02 mg/g), epicatechin gallate (ECG) (1.76 ± 0.62 mg/g), and epigallocatechin (EGC) (0.21 ± 0.23 mg/g) (Figure 1, Table 7). These findings indicate that EGCG was the predominant catechin in the fermented Miang samples.

Previous studies have similarly reported that the major catechins commonly found in *C. sinensis* are EC, ECG, EGC, and EGCG [20,21]. In this study, the HPLC method was used to identify and quantify catechins in FM leaves and demonstrated suitability for analyzing catechin content in cosmetic and dermatological formulations. This supports its use for assessing catechin levels in topical preparations [22,23].

The variation in catechin content among FM samples may be influenced by several factors, including leaf maturity, fermentation duration, drying temperature, and extraction method [24]. The results suggest that the 4-month fermentation period favored catechin accumulation, and the extraction temperature range of 77–80 °C was optimal for obtaining high yields of phenolic compounds [25,26].

EGCG and ECG are known for their strong antioxidant properties and diverse pharmacological activities, including anti-inflammatory, antimicrobial, antiviral, antiallergenic, and anticancer effects [27]. Among the catechins, EC exhibited the most potent radical-scavenging activity [15]. Catechins have also been reported to promote collagen synthesis and inhibit the activity of matrix metalloproteinase (MMP) enzymes, thereby reducing photoaging-related skin damage [25]. Moreover, black tea extracts rich in catechins have demonstrated photoprotective effects against UVB-induced sunburn and epidermal thickening [26].

Consistent with these findings, our previous study showed that FM extract exhibited strong superoxide (O_2_^−^) scavenging activity with an IC_50_ value of 0.038 mg/mL, along with inhibitory effects on tyrosinase and hyaluronidase enzymes, as well as antiglycation properties [6]. Importantly, catechins have been shown to be safe for topical application and are widely utilized in the development of antioxidant-based cosmetic products [28].

### 3.3. Lactobacillus Content in Fermented Miang

A total of sixteen Miang (FM) samples were analyzed for *Lactobacillus* content, as summarized in Table 8. The samples included three 1-month fermented young leaf samples, three 2-month fermented young leaf samples, one 2-month fermented mature leaf sample, and seven 12-month fermented mature leaf samples. Two fresh (unfermented) Miang leaf samples were used as controls.

The extraction yield of FM samples ranged from 4.40% to 8.74% (*w*/*w*) (Table 8), supporting their potential application as cosmetic ingredients. Viable *Lactobacillus* counts ranged from 10^4^ to 10^8^ CFU/g, which may contribute to probiotic benefits desirable in cosmetic formulations. No *Lactobacillus* colonies appeared in the control (fresh leaf) samples. The identified *Lactobacillus* species—*L. lindneri*, *L. acidophilus*, *L. lactis* subsp. *lactis*, *L. delbrueckii* subsp. *delbrueckii*, *L. brevis*, and *L. plantarum* are of interest due to their established relevance in skincare and cosmetic efficacy.

Higher bacterial counts appeared in young leaves fermented for at least two months and in mature leaves fermented for twelve months. These differences in *Lactobacillus* growth, influenced by fermentation time and conditions, are particularly significant for cosmetic applications, as such microbial populations can affect product efficacy and safety. Variation in *Lactobacillus* between samples may result from differences in fermentation methods and environmental conditions at the source, which may impact the consistency and quality of cosmetic formulations.

The presence of *Lactobacillus* species in fermented tea leaves is of particular interest for cosmetic applications, as these bacteria can enhance the bioavailability of active compounds, boost antioxidant activity, and provide probiotic benefits to skincare products. Such characteristics make fermented tea extracts promising candidates for skin care formulations, especially those aimed at anti-aging, hydration, and skin barrier repair [7], supporting their relevance in developing innovative cosmetics.

### 3.4. Prototype Formulations and Stability

The antioxidant activity of each formulation was evaluated using a sample concentration of 1 mg/mL, with the DPPH solution serving as a standard control. A higher percentage of inhibition indicated stronger antioxidant activity.

The liquid formulations (FM Liquid Soap and FM Shampoo) appeared as clear, pale-yellow products with a characteristic fermented Miang odor and exhibited no phase separation. Both demonstrated good cleansing performance. The FM Liquid Soap showed a pH of 6.23 ± 0.02 and % inhibition of 7.85 ± 0.34, whereas the FM Shampoo exhibited a pH of 7.08 ± 0.02 and % inhibition of 9.13 ± 0.52.

The FM Glycerin Soap Bar was a hard, pale yellow–to–light red bar with a typical fermented odor, moderate foaming ability, and a moisturizing effect on the skin. It had a pH of 9.95 ± 0.00 and % inhibition of 8.55 ± 1.50. The FM Opaque Soap Bar was off-yellow, harder in texture, and demonstrated better foaming and cleansing efficiency than the glycerin-based bar, with a pH of 9.20 ± 0.02 and % inhibition of 8.40 ± 1.20. All formulations complied with acceptable physicochemical characteristics for cosmetic cleansing products.

Accelerated stability testing was performed under various storage conditions: 4 °C, 45 °C, room temperature (25 ± 2 °C), and cyclic hot–cold (H&C) conditions. Using samples stored at 4 °C as the control, comparisons with other conditions revealed no significant changes in color, phase separation, or pH throughout the testing period. Minor textural changes were observed in the Glycerin Soap Bar and Opaque Soap Bar, accompanied by slight variations in % inhibition, likely due to differences in soap base composition.

**Figure 2:**
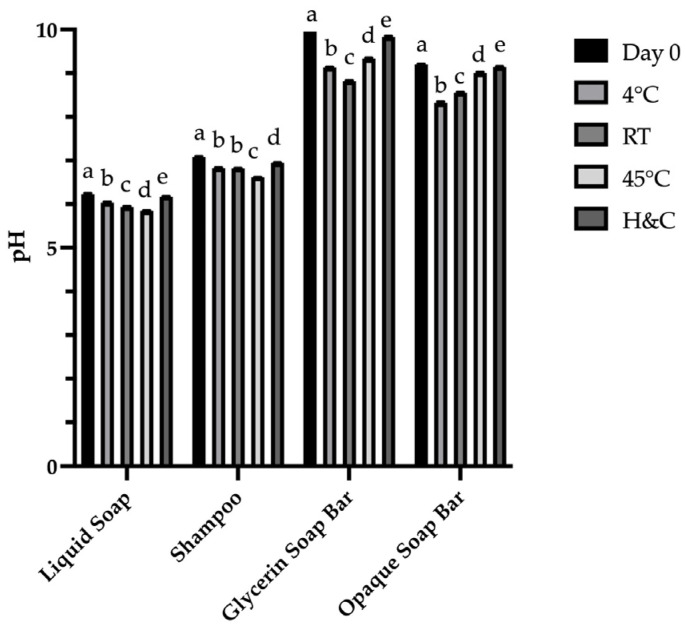
pH of cosmetic products after 3 months of storage under different conditions. Notes: The results are presented as the mean ± SD. (n = 3). In each assay, the presence of different superscript letters (a–e) in the same column indicates a significant difference according to Tukey’s test (*p* < 0.05).

**Figure 3:**
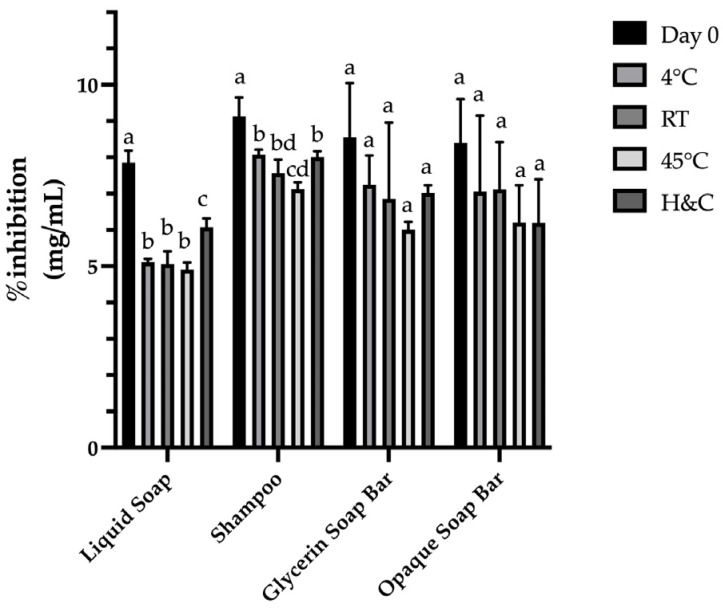
Percent DPPH inhibition of cosmetic products after 3 months of storage. Notes: The results are presented as the mean ± SD. (n = 3). In each assay, the presence of different superscript letters (a–d) in the same column indicates a significant difference according to Tukey’s test (*p* < 0.05).

Overall, both the FM Shampoo and FM Liquid Soap demonstrated the best physical and bioactive stability, indicating their potential as prototype antioxidant cleansing products. Nevertheless, long-term stability testing should be performed to accurately predict shelf life and ensure product performance over time. The results of physicochemical and bioactivity stability assessments are presented in Figure 2 and Figure 3, and Table 9. These formulations represent preliminary prototype systems designed to evaluate the feasibility of incorporating fermented Miang extract into cosmetic products. The physicochemical properties observed are consistent with typical rinse-off formulations; however, further studies are required to assess dermatological safety (e.g., irritation and patch testing), microbial stability, and comparison with commercial benchmarks. Therefore, the present work serves as a foundational step toward the development of fermented plant-based cosmeceuticals. Although the DPPH assay is widely used for evaluating radical scavenging activity, it represents only a single mechanism of antioxidant action. In this study, DPPH was primarily employed as a comparative tool to assess the relative effects of fermentation time and formulation stability under controlled conditions. Future studies should incorporate additional assays, such as ABTS and FRAP, as well as comparisons with standard antioxidants and non-fermented tea samples, to provide a more comprehensive evaluation of antioxidant capacity.

It should be noted that catechins are known to be unstable under neutral to alkaline conditions, where oxidation, epimerization, and polymerization reactions may occur [29,30]. In this study, some formulations exhibited pH values in the range of 6–9, which may contribute to partial degradation of catechins. However, measurable antioxidant activity was still observed, which may be attributed to the presence of residual intact catechins and/or the formation of transformation products that retain redox activity. These results suggest that antioxidant performance in the formulations may arise from a combination of native and modified phenolic compounds. Nevertheless, direct quantification of catechin content in the finished formulations would provide more definitive insight into stability and should be considered in future studies.

At higher pH conditions, partial ionization of phenolic hydroxyl groups may reduce the efficiency of catechins as primary antioxidants. However, antioxidant activity in such systems may still occur through alternative mechanisms, including electron transfer and secondary antioxidant pathways. In addition, the complex mixture of phenolic compounds and fermentation-derived metabolites present in fermented Miang extract may contribute collectively to the observed antioxidant activity.

## 4. Conclusions

Fermented Miang demonstrated significant functional potential, as indicated by its high total phenolic content, total flavonoid content, antioxidant capacity, and the presence of beneficial *Lactobacillus* species without detectable pathogens. Among all samples, the four-month fermented extract (FMB) exhibited the highest levels of phenolic and flavonoid compounds and the strongest antioxidant activity.

Storage temperature significantly influenced the antioxidant stability of both FM extracts and formulations. Samples stored at 4 °C retained higher antioxidant activity compared with those kept at 45 °C or room temperature, whereas prolonged storage (beyond three months) resulted in a gradual reduction of antioxidant potency. When compared with the standard antioxidant Trolox, nine FM samples displayed lower antioxidant activity, though they remained biologically active and suitable for cosmetic applications.

The fermented Miang extract also served as a natural source of *Lactobacillus* species, consistent with previous findings reporting the presence of numerous lactic acid bacterial strains in fermented tea products. These microorganisms, together with their lysates or metabolites, could be utilized in probiotic or bioactive phytochemical-based cosmetic formulations.

These findings suggest that fermented Miang could serve as a promising natural source of catechin-rich extracts for antioxidant-based cosmetic formulations and probiotic-inspired skincare products.

## Figures and Tables

**Table 1:** Transparent FM soap bar formulation.

Name	% (*w*/*w*)	Function
FM extract	5.00	Active
Rice bran essence	0.50	Fragrance
Glycerin soap base	94.50	Base

**Table 2:** Opaque FM soap bar formulation.

Name	% (*w*/*w*)	Function
FM extract	5.00	Active
Coconut oil	25.50	Soap base component
Palm oil	25.50	Soap base component
Sesame oil	6.40	Soap base component
Olive oil	3.20	Soap base component
Castor oil	3.20	Soap base component
Sodium hydroxide	9.60	Saponifying agent
Distilled water	21.00	Diluent
Rice bran essence	0.60	Fragrance

**Table 3:** FM liquid soap formulation.

Name	% (*w*/*w*)	Function
FM extract	5.00	Active
Ammonium lauryl sulfate	35.00	Surfactant
Cocamidopropyl betaine	7.00	Surfactant
Lauryl glucoside 1200	6.00	Surfactant
Ammonium chloride	0.70	Viscosity modifier
Preservative	0.60	Preservative
Natural color	1.00	Colorant
Rice bran essence	0.50	Fragrance
Distilled water	44.20	Diluent

**Table 4:** FM shampoo formulation.

Phase	Name	% (*w*/*w*)	Function
A	Sodium Laureth Sulfate	38.00	Surfactant
	Cocamidopropyl betaine	6.00	Cleanser
	DEA	1.65	Viscosity inducer
	Polyquaternium-7	2.60	Conditioning
	Bis(C13–15 Alkoxy) PG–Amodimethicone	0.20	Protection for damaged hair
B	Distilled water	34.60	Solvent
	NaCl	0.70	Thickener
C	Distilled water	10.0	Solvent
	Polyquaternium-67	0.25	Antistatic
D	FM extract	5.00	Active
E	Preservative	1.00	Preservative

**Table 5:** Effect of fermentation time on total phenolic content (TPC), total flavonoid content (TFC), and antioxidant activity of fermented Miang extracts.

Sample	Fermentation Time (Months)	TPC (mg GAE/100 g)	TFC (mg QE/100 g)	IC_50_ (mg/mL)	% Inhibition (1 mg/mL)
FMA	2	108.46 ± 0.79 ^a^	22.30 ± 0.11 ^a^	10.76 ± 0.47 ^a^	11.13 ± 0.54 ^a^
FMB	4	263.22 ± 2.01 ^b^	74.66 ± 2.65 ^b^	4.44 ± 0.00 ^b^	15.62 ± 0.04 ^b^
FMC	6	85.68 ± 2.00 ^c^	17.19 ± 1.03 ^c^	13.57 ± 0.00 ^c^	9.80 ± 0.76 ^c^

Note: GAE = gallic acid equivalents; QE = quercetin equivalents. The results are presented as the mean ± SD. (n = 3). In each assay, the presence of different superscript letters (a–c) in the same column indicates a significant difference according to Tukey’s test (*p* < 0.05).

**Table 6:** Stability of fermented Miang extract (FMB) in terms of % inhibition (at 1 mg/mL) and pH over a 3-month storage period under various conditions.

Stability	Month	4 °C	45 °C	RT (25 ± 2 °C)	H&C
% Inhibition	0	-	-	15.62 ± 0.04 ^a^	-
	1	14.22 ± 0.15 ^a^	12.25 ± 0.24 ^a^	14.10 ± 0.10 ^b^	12.24 ± 0.34 ^d^
	3	13.62 ± 0.26 ^b^	13.10 ± 0.30 ^b^	13.50 ± 0.11 ^c^	-
pH	0	-	-	6.02 ± 0.03 ^a^	-
	1	NC ^a^	NC ^a^	NC ^a^	5.93 ± 0.01 ^d^
	3	5.58 ± 0.01 ^b^	5.34 ± 0.02 ^b^	5.52 ± 0.01 ^b^	-

Notes: NC = no change; RT = room temperature; H&C = cyclic hot–cold condition. The results are presented as the mean ± SD. (n = 3). In each assay, the presence of different superscript letters (a–c) in the same column indicates a significant difference according to Tukey’s test (*p* < 0.05). For the hot and cold stability tests, the superscript letter (d) indicates a statistically significant difference, as determined by the paired t-test (*p* < 0.05).

**Table 7:** Catechin composition of fermented Miang extract (FMB) determined by HPLC.

No.	Compound	Content (mg/g Dry Sample)
1	Epicatechin (EC)	2.23 ± 0.02 ^a^
2	Epigallocatechin gallate (EGCG)	7.00 ± 0.93 ^b^
3	Epigallocatechin (EGC)	0.21 ± 0.23 ^c^
4	Epicatechin gallate (ECG)	1.76 ± 0.62 ^a^
5	Catechin (C)	2.41 ± 0.02 ^a^

Note The results are presented as the mean ± SD. (n = 3). In each assay, the presence of different superscript letters (a–c) in the same column indicates a significant difference according to Tukey’s test (*p* < 0.05).

**Table 8:** Physical characteristics and extraction yield of fermented Miang (FM) samples.

Sample	Maturity and Fermentation Duration	Appearance (Before Fermentation)	Appearance (After Fermentation)	% Yield (*w*/*w*)	Lactic Acid Bacteria (CFU/g)
PM-fresh	Fresh leaf, mature	Dark green, thick leaf	Brown	7.31	Not found
PM-001	Young leaf, 1 month	Green-brown leaf	Brown	4.88	9.3 × 10^6^
PM-002	Mature leaf 12 months	Brown-green leaf	Brown	5.74	4.9 × 10^4^
PM-003	Young leaf, 2 months	Brown-green leaf	Brown	5.62	6.9 × 10^7^
PM-004	Mature leaf, 2 months	Green, brown leaf	Green	6.02	3.1 × 10^7^
PM-005	Young leaf, 2 months	Green brown leaf	Green brown	8.43	1.1 × 10^7^
PM-006	Mature leaf, 12 months	Brown leaf	Brown	4.90	6.6 × 10^6^
PM-007	Young leaf, 2 months	Green-brown leaf	Green brown	8.74	8.4 × 10^6^
PM-008	Mature leaf, 12 months	Brown leaf	Green brown	5.31	5.6 × 10^6^
PM-009	Young leaf, 1 month	Brown leaf	Brown	4.40	5.4 × 10^6^
PM-010	Young leaf, 1 month	Brown leaf	Brown	4.62	7.3 × 10^6^
MJ-fresh	Fresh leaf, mature	Light-green leaf	Brown	5.81	Not found
MJ-001	Mature leaf, 12 months	Green, brown leaf	Light brown	6.46	9.3 × 10^7^
MJ-002	Mature leaf, 12 months	Green, brown leaf	Dark brown	6.82	1.1 × 10^8^
MJ-003	Mature leaf, 12 months	Brown leaf	Brown	6.03	6.6 × 10^6^
MJ-004	Mature leaf, 12 months	Green, brown leaf	Green, brown	4.89	9.6 × 10^6^

Notes: PM = samples collected from Pa-Miang village; MJ = samples collected from Mae-Jam village; CFU = colony-forming units.

**Table 9:** Stability of cosmetic products containing fermented Miang extract (FMB) under various storage conditions.

Product	Condition (After 3 Months)	Physical Characteristics	pH	% Inhibition (1 mg/mL DPPH)
Liquid Soap	Day 0	Clear, pale yellow color; characteristic FM odor; no phase separation; good cleansing effect	6.23 ± 0.02 ^a^	7.85 ± 0.34 ^a^
	4 °C	NC	6.03 ± 0.02 ^b^	5.11 ± 0.10 ^b^
	RT	Reduced viscosity; clear yellow color; no phase separation; good cleansing effect	5.93 ± 0.02 ^c^	5.05 ± 0.35 ^b^
	45 °C	Slight decrease in viscosity	5.84 ± 0.02 ^d^	4.91 ± 0.20 ^b^
	H&C	NC	6.17 ± 0.01 ^e^	6.08 ± 0.24 ^c^
Shampoo	Day 0	Clear, pale yellow solution; characteristic FM odor; no phase separation; good cleansing effect	7.08 ± 0.02 ^a^	9.13 ± 0.52 ^a^
	4 °C	NC	6.82 ± 0.01 ^b^	8.08 ± 0.13 ^b^
	RT	NC	6.81 ± 0.01 ^b^	7.55 ± 0.38 ^b,d^
	45 °C	NC	6.62 ± 0.01 ^d^	7.12 ± 0.20 ^c,d^
	H&C	NC	6.94 ± 0.01 ^e^	8.01 ± 0.16 ^b^
Glycerin Soap Bar	Day 0	Yellow to pale red hard soap; characteristic FM odor; medium foam; moisturizing effect	9.95 ± 0.00 ^a^	8.55 ± 1.50 ^a^
	4 °C	NC	9.13 ± 0.02 ^b^	7.25 ± 0.81 ^a^
	RT	NC	8.82 ± 0.02 ^c^	6.85 ± 2.10 ^a^
	45 °C	Texture changed	9.33 ± 0.02 ^d^	6.01 ± 0.22 ^a^
	H&C	Texture changed	9.83 ± 0.02 ^e^	7.02 ± 0.21 ^a^
Opaque Soap Bar	Day 0	Off-white to yellow hard soap; characteristic FM odor; good foaming and cleansing effect	9.20 ± 0.02 ^a^	8.40 ± 1.20 ^a^
	4 °C	Slight color change	8.32 ± 0.03 ^b^	7.05 ± 2.10 ^a^
	RT	Slight color change	8.55 ± 0.02 ^c^	7.10 ± 1.31 ^a^
	45 °C	Texture changed	9.00 ± 0.02 ^d^	6.21 ± 1.03 ^a^
	H&C	Texture changed	9.14 ± 0.02 ^e^	6.20 ± 1.20 ^a^

Notes: NC = no change; H&C = cyclic hot–cold condition; RT = Room temperature. The results are presented as the mean ± SD. (n = 3). In each assay, the presence of different superscript letters (a–e) in the same column indicates a significant difference according to Tukey’s test (*p* < 0.05).

## Data Availability

The original contributions presented in this study are included in the article. Further inquiries can be directed to the corresponding author.

## Abbreviations

The following abbreviations are used in this manuscript:

BAM	Bacteriological analytical manual
C	Catechin
CFU	colony-forming units
DPPH	2,2-diphenyl-1-picrylhydrazyl
EC	Epicatechin
ECG	Epicatechin gallate
EGCG	Epigallocatechin gallate
FM	Fermented Miang
H&C	Cyclic hot–cold condition
HPLC	High-performance liquid chromatography
MJ	Samples collected from Mae-Jam village
MRS	Man, Rogosa, and Sharpe
NC	No change
PM	Samples collected from Pa-Miang village
QE	Quercetin equivalents
RT	Room temperature
TFC	Total flavonoid content
TPC	Total phenolic content
TSB	Tryptic soy broth
